# Quality of life in subjects with upper- and lower-limb spasticity treated with incobotulinumtoxinA

**DOI:** 10.1186/s12955-020-01304-4

**Published:** 2020-03-04

**Authors:** Klemens Fheodoroff, Tiina Rekand, Luisa Medeiros, Peter Koßmehl, Jörg Wissel, Djamel Bensmail, Astrid Scheschonka, Birgit Flatau-Baqué, Olivier Simon, Dirk Dressler, David M. Simpson

**Affiliations:** 1 Gailtal-Klinik, Hermagor, Austria; 2 Haukeland University Hospital, Bergen, Norway; 3 Centro Hospitalar de Lisboa Central, Lisbon, Portugal; 4 Kliniken Beelitz GmbH, Beelitz-Heilstätten, Beelitz, Germany; 5 Vivantes Hospital Spandau, Berlin, Germany; 6 Raymond-Poincaré Hospital, AP-HP, University of Versailles Saint Quentin, Garches, France; 7 Merz Pharmaceuticals GmbH, Frankfurt am Main, Germany; 8 Formerly of Merz Pharmaceuticals GmbH, Frankfurt am Main, Germany; 9 Hannover Medical School, Hannover, Germany; 10 Icahn School of Medicine at Mount Sinai, New York, New York, USA

**Keywords:** Botulinum neurotoxin, IncobotulinumtoxinA, Long-term care, Lower limb, Quality of life, Spasticity, Spasticity management, Upper limb

## Abstract

**Background:**

We evaluated quality of life among subjects with upper- and lower-limb spasticity who received escalating doses of incobotulinumtoxinA (total body doses up to 800 U) in the prospective, single-arm, dose-titration TOWER study.

**Methods:**

In this exploratory trial, subjects (*N* = 155; 18–80 years of age) with upper- and lower-limb spasticity due to cerebral causes who were deemed to require total body doses of up to 800 U incobotulinumtoxinA received three consecutive injection cycles of incobotulinumtoxinA (400, 600, and up to 800 U), each with 12 to 16 weeks’ follow-up. QoL was assessed using the EuroQol 5-dimensions questionnaire, three-level (EQ-5D), before and 4 weeks post-injection in each injection cycle and at the end of injection cycle 3.

**Results:**

The mean EQ-5D visual analog scale scores of 155 participants continuously improved from study baseline to 4 weeks post-injection in all injection cycles (mean [standard deviation] change 6.7 [14.1], 9.6 [16.3], and 8.6 [17.0] for injection cycles 1, 2, and 3, respectively; *p* < 0.0001 for all, paired sample t-test). In general, among those with a change in the EQ-5D rating of their condition, the proportion of subjects with ‘improvement’ was greater than that with ‘worsening’ for individual EQ-5D dimensions across all injection cycles. At the end of injection cycle 3, the proportion of subjects rating their condition as ‘normal’ increased from study baseline for all dimensions, and there was a ≥ 46% reduction in the proportion of subjects with a rating of ‘severe impairment’.

**Conclusion:**

These preliminary results suggest that escalating incobotulinumtoxinA doses up to 800 U are associated with improvement in quality of life ratings in subjects with multifocal upper- and lower-limb spasticity, and form a basis for future comparator studies.

**Trial registration:**

ClinicalTrials.gov, NCT01603459. Date of registration: May 22, 2012.

## Introduction

Disabling spasticity of the upper and lower limbs is a common and often painful complication of stroke and results in marked limitations of subjects’ mobility and their ability to perform routine daily tasks, such as dressing and personal hygiene [1–5]. These effects have an impact on subjects’ quality of life (QoL), with many subjects reporting dependence on family and carers, social isolation, and depression [1, 2, 6, 7]. Improvement in QoL is often a key goal for subjects with spasticity [1]; QoL measures should therefore play a vital role in the assessment of clinical interventions.

Botulinum neurotoxin type A (BoNT-A) formulations are recommended in general spasticity guidelines for the treatment of focal spasticity of the upper and lower limbs [8, 9]. The efficacy and safety of treatment with BoNT-A formulations in subjects with focal spasticity are well established [10–19]. However, treating multifocal upper- or lower-limb spasticity may require total doses of BoNT-A higher than those currently approved, in order to meet subjects’ clinical needs and goals of rehabilitation therapy [20].

Previous data have indicated the immunologic and toxicologic safety of incobotulinumtoxinA doses up to 1200 mouse units in subjects with spasticity or dystonia [21]. Recommendations of incobotulinumtoxinA for upper-limb spasticity include ≤400 U per session in the United States and ≤ 500 U per session in the European Union, no more frequently than every 12 weeks [22, 23]. The prospective, single-arm, dose-titration TOWER study was designed to assess the safety and efficacy of escalating incobotulinumtoxinA doses (total body doses of 400 U, 600 U, and up to 800 U) in adults with multifocal spasticity of the upper and lower limbs, on the same body side, as a result of stroke or other cerebral causes [20]. With regards to safety, the incidence of treatment-related adverse events did not increase with escalating incobotulinumtoxinA doses up to 800 U. Dose escalation improved muscle tone, with mean improvements in Resistance to Passive Movement Scale (REPAS) [24] scores at 4 weeks post-injection of − 4.6, − 5.9, and − 7.1 for consecutive injection cycles with 400 U, 600 U, and up to 800 U, respectively (all *p* < 0.0001, Student’s t-test for paired samples) [20].

While the safety and efficacy of BoNT-A treatments are well reported, data on subjects’ QoL following BoNT-A treatment remain limited and largely inconclusive [1, 3, 25], with extremely limited data available to date on QoL following combined treatment of upper-and lower-limb spasticity [1]. Furthermore, few studies report baseline scores for QoL in untreated subjects with spasticity [3, 4], leading to a lack of normative data for comparison. In order to address this knowledge gap and to better understand the effects of escalating incobotulinumtoxinA treatment on subjects’ QoL, we report QoL outcomes based on the EuroQol 5-dimensions questionnaire, three-level (EQ-5D) [26, 27], following incobotulinumtoxinA treatment of upper- and lower-limb spasticity in the TOWER study.

## Methods

### Study design

TOWER (The Titration study in lOWer and uppER limb spasticity; NCT01603459) was a prospective, single-arm, multicenter, non-randomized dose-titration study investigating the safety and efficacy of incobotulinumtoxinA in subjects with upper- and lower-limb spasticity who were deemed by the investigator to require total body doses of 800 U incobotulinumtoxinA. The study was conducted at 30 centers in Europe and North America. To minimize potential bias of subject-rated outcomes, subjects were blinded to which dose they were receiving during which cycle. Study details, safety and results from selected efficacy variables have been published elsewhere [20].

In brief, the study comprised three consecutive, flexible, 12–16-week injection cycles, in which the following escalating doses of incobotulinumtoxinA (BoNT-A free from complexing proteins, Xeomin®, Merz Pharmaceuticals GmbH) were administered by intramuscular injection in the same body side on the first day of each injection cycle: cycle 1, a fixed total body dose of 400 U into the upper limb or lower limb only, or split between both; cycle 2, a fixed total body dose of 600 U into the upper limb or lower limb only, or split between both; cycle 3, a total body dose of 800 U split between both upper and lower limbs (maximum dose of 600 U per limb) or a lower total dose of between 600 and 800 U, if 800 U was not indicated for clinical or safety reasons.

The study was conducted in accordance with the principles of the Declaration of Helsinki. The protocol was approved by the local institutional review boards and independent ethics committees.

### Subjects

Subjects eligible to participate in the study were male or female and 18–80 years of age with chronic (≥ 12 weeks since the last event leading to spasticity on the side of the body with the selected target clinical pattern diagnosed by a healthcare professional) upper- and lower-limb spasticity of the same body side due to cerebral causes, and were considered by the investigator to require total body doses of 800 U incobotulinumtoxinA during the course of the study. Subjects with bilateral symptoms due to cerebral or brainstem lesions were eligible if they agreed to be treated on only one side of the body. The area of the target clinical pattern selected for treatment was required to have an Ashworth Scale score ≥ 2 and a Disability Assessment Scale (DAS) score ≥ 2 (upper limb). Study entry exclusion criteria included: spinal lesions; neurological conditions associated with neuromuscular dysfunction; fixed contracture or muscle hypertonia other than spasticity in the joint associated with the selected target clinical pattern; severe atrophy of muscles associated with the target clinical pattern; prior or planned surgery of the target limb (within 8 weeks) or muscle; treatment with intrathecal baclofen or antispasticity medication with peripheral muscle relaxants within 2 weeks prior to screening; change in antidepressant medication within 4 weeks prior to screening or within the screening period; change in antispastic medication with centrally acting muscle relaxants within 2 weeks prior to screening or within the screening period; infection of planned injection sites; generalized muscle disorders or any other peripheral neuromuscular dysfunction; rheumatic disease in the limbs of the targeted body side; adverse reaction of severe intensity to BoNT-A or BoNT-B lasting > 1 week within previous 12 months; international normalized ratio (INR) value > 1.5 and/or a partial thromboplastin time value of > 1.5-times the upper limit of normal; and forced expiratory volume in 1 s (FEV_1_) < 70%. Eligibility criteria for injection cycles 2 and 3 included clinical justification for doses of incobotulinumtoxinA ≥600 U (confirmed by a neurologist or rehabilitation physician), absence of infection at the planned injection site, absence of severe weakness of the target muscle that would preclude injection of incobotulinumtoxinA, INR < 1.5, and FEV_1_ ≥ 60%.

All subjects were required to provide written informed consent. The subject-reported outcomes, which were evaluated to address the impact of treatment on subject QoL, are detailed below.

### Assessment of quality of life

Quality of life was assessed as an efficacy variable in the TOWER study. Subjects completed the three-level version of EQ-5D [26, 27] at the injection baseline visit of each injection cycle, at 4 weeks post-injection ±3 days in each injection cycle, and at the end-of-cycle-3 visit (end of study), giving a total of 7 assessments.

Subjects rated their current state of health on a quantitative visual analog scale (VAS) from 0 (worst) to 100 (best). For each dimension of the EQ-5D descriptive system (Mobility, Self-care, Usual activities, Pain/discomfort and Anxiety/depression), subjects also selected the statement that best described their health state on that day: no problems reported (normal condition, score 1); some problems reported (moderate impairment, score 2); and extreme problems reported (severe impairment, score 3).

### Statistical analysis

As this was an exploratory trial, no formal sample size calculation was performed. The efficacy analyses are based on the full analysis set (FAS), which included all subjects who received ≥1 dose of the study medication. Variables were analysed by descriptive statistics. Analysis of EQ-5D scores was performed using observed cases. Mean changes in EQ-5D VAS score from the study baseline to 4 weeks post-treatment in each injection cycle were assessed by the Student’s t-test (two-sided) for paired samples. Changes in EQ-5D dimension data from each injection cycle baseline to 4 weeks post-injection were analyzed using frequency tables. In addition, we describe the frequencies of EQ-5D dimension scores at study baseline and the end of injection cycle 3.

## Results

### Subjects and exposure

A total of 155 subjects participated and were treated in the study between May 2012 and September 2014, and were included in the FAS (Additional file 1: Fig. S1). Eighteen (11.6%) subjects discontinued the study, with three subjects discontinuing after injection in injection cycle 1, 12 subjects after injection in cycle 2, and three subjects after injection in cycle 3. Details of the reasons for study discontinuation have been published previously [20].

Subject characteristics and baseline demographics are summarized in Table 1. The subjects had a mean (standard deviation [SD]) age of 53.7 (13.1) years and the majority were male (67.1%). Cerebral vascular disorders were the cause of spasticity for 138 (89.0%) cases, with 85.2% of subjects having cerebral stroke as the cause. Median time since the diagnosis of the event leading to spasticity of the right and left body sides was 46.5 and 61.4 months, respectively. At study baseline, the mean (SD) REPAS score for assessment of muscle tone of the treated body side was 24.8 (6.7) and the mean (SD) score for all functional domains of the DAS ranged from 2.6 (0.5) to 2.8 (0.4).

**Table 1:** Subject demographics and baseline characteristics (FAS)

Characteristic	Subjects *N* = 155
Age (years), mean (SD)	53.7 (13.1)
Sex, *n* (%)	
Male	104 (67.1)
Race, *n* (%)	
White	129 (83.2)
Black/African American	4 (2.6)
Other	3 (1.9)
Missing	19 (12.3)
BMI (kg/m^2^), mean (SD)	27.5 (4.9)
Cause of spasticity, *n* (%)	
Ischemic stroke	87 (56.1)
Hemorrhagic stroke	45 (29.0)
Traumatic brain injury	11 (7.1)
Other cerebral vascular disorders	6 (3.9)
Brain tumor	4 (2.6)
Cerebral palsy	2 (1.3)
Time since diagnosis of event leading to spasticity (months), median (range)	
Left body side; *n* = 81	61.4 (2.8–428.9)
Right body side; *n* = 68	46.5 (3.7–372.8)
REPAS^a^ score at study baseline, mean (SD)	24.8 (6.7)
DAS^b^ score at study baseline, mean (SD)	
Principle therapeutic target	2.6 (0.5)
Hygiene	2.6 (0.5)
Dressing	2.6 (0.5)
Limb position	2.6 (0.5)
Pain	2.8 (0.4)

^a^REPAS score of the treated body side consisting of the sum scores for 13 items, each rated using the Ashworth Scale from 0 to 4, ranging from 0 (no resistance to passive movement for any of the items) to 52 (limbs rigid to passive movement for all items)

^b^DAS score, ranging from 0 (no disability) to 3 (severe disability, normal activities limited)

*BMI* body mass index, *DAS* Disability Assessment Scale, *FAS* full analysis set, *REPAS* Resistance to Passive Movement Scale, *SD* standard deviation

All subjects had spasticity of both the upper and lower limb. One subject had spasticity of both upper limbs and two subjects had spasticity of both lower limbs. The majority of subjects (85.8%) had previously received treatment with BoNT (type A or B). Median (range) time since the most recent injection of BoNT was 4.4 months (2.7–93.9 months). Depression was reported for 34.8% of subjects and anxiety was reported for 11.0% of subjects. In total, 43.9 and 20.6% of subjects were receiving concomitant psychoanaleptics and psycholeptics, respectively.

Seventy-one (45.8%) subjects were treated on the right side of the body and 84 (54.2%) subjects were treated on the left side. Most subjects received the scheduled dose in each injection cycle (91.0% [141/155] received 400 U in cycle 1; 90.8% [138/152] received 600 U in cycle 2; and 82.9% [116/140] received 800 U in cycle 3). A total of 140 (90.3%) subjects received all three injections.

### Assessment of quality of life

#### EQ-5D VAS

**Fig. 1:**
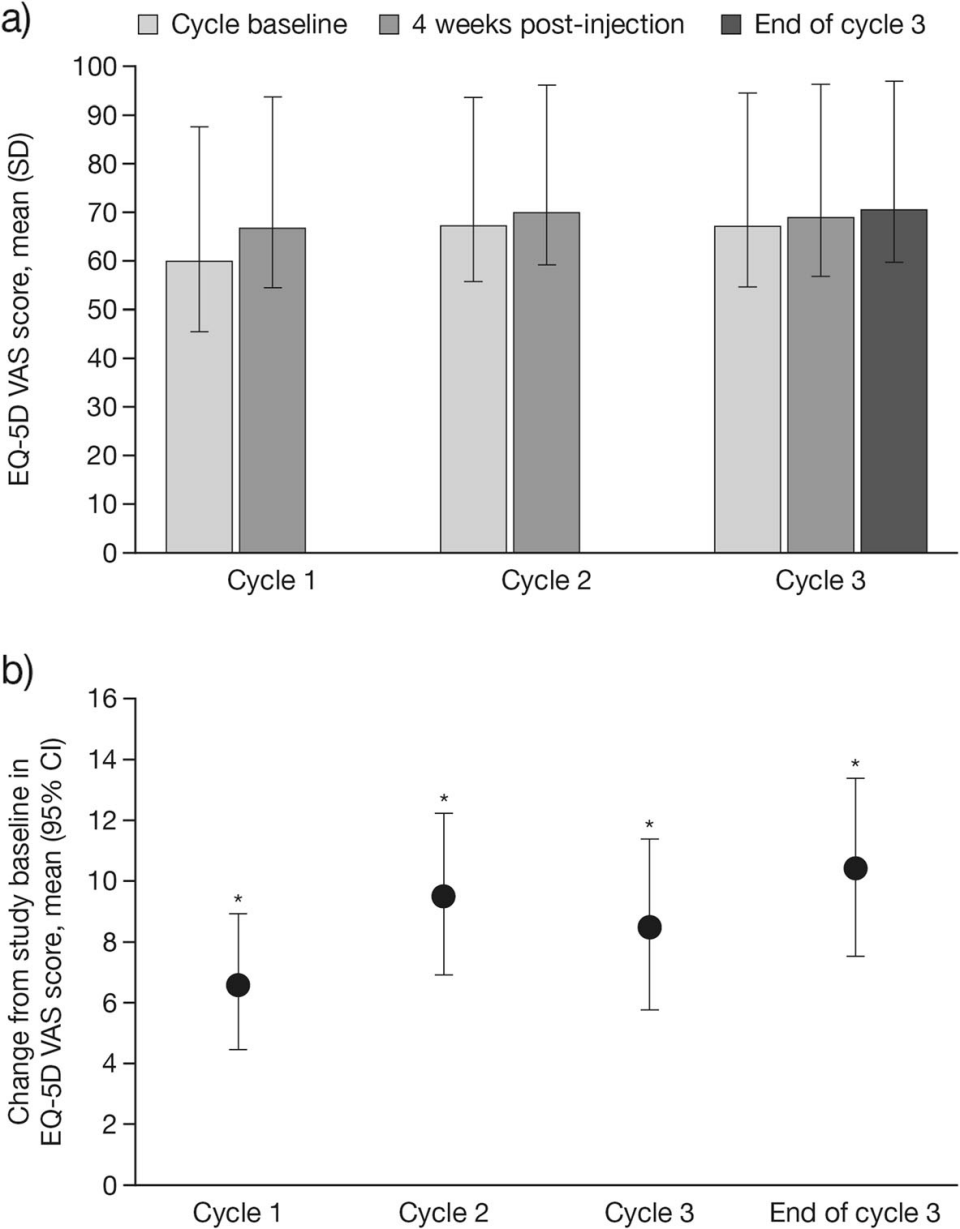
**a** Mean (SD), and **b** mean (95% CI) change in EQ-5D VAS scores (FAS). a) Time points: at injection cycle baseline and 4 weeks post-injection in each injection cycle and at the end of injection cycle 3. b) Time points: from the study baseline to 4 weeks post-injection in each injection cycle and to the end of injection cycle 3. Positive values indicate improvement. *p < 0.0001 compared with the study baseline visit in injection cycle 1, Student’s t-test for paired samples. *CI*, confidence interval; *EQ-5D*, EuroQol 5-dimensions questionnaire, three-level; *FAS*, full analysis set; *SD*, standard deviation; *VAS*, visual analog scale

Compared with study baseline, there was an improvement in mean EQ-5D VAS score 4 weeks post-injection in cycle 1, which was sustained with subsequent injections. The mean (SD) EQ-5D VAS score improved from 59.9 (18.9) at the study baseline to 66.7 (17.6) 4 weeks post-injection in cycle 1, and improved further to 70.5 (16.7) at the end of cycle 3 (Fig. 1a), corresponding to a mean (95% confidence interval [CI]) change of 10.5 (7.6, 13.4) from study baseline to the end of cycle 3 (*p* < 0.0001, Student’s t-test for paired samples; Fig. 1b). The mean (SD) change in EQ-5D VAS score from the study baseline to 4 weeks post-treatment was 6.7 (14.1) in cycle 1, 9.6 (16.3) in cycle 2, and 8.6 (17.0) in cycle 3 (*p* < 0.0001 for all versus study baseline, Student’s t-test for paired samples).

#### EQ-5D dimension ratings

Among those subjects who had a change in the rating of their condition at 4 weeks post-injection, a greater proportion of subjects had ‘improvement’ than ‘worsening’ for all dimensions across all injection cycles, with the exception of Self-care in injection cycle 2 (Table 2).

**Table 2:** Change in EQ-5D dimensions from injection cycle baseline to 4 weeks post-injection (FAS)

Dimension	Injection cycle	*N*	Nobs	Improvement, *n* (%)	No change, *n* (%)	Worsening, *n* (%)
Mobility	1	155	155	18 (11.6)	135 (87.1)	2 (1.3)
	2	152	149	11 (7.2)	136 (89.5)	2 (1.3)
	3	140	138	6 (4.3)	128 (91.4)	4 (2.9)
Self-care	1	155	155	19 (12.3)	123 (79.4)	13 (8.4)
	2	152	148	7 (4.6)	127 (83.6)	14 (9.2)
	3	140	138	11 (7.9)	121 (86.4)	6 (4.3)
Usual activities	1	155	155	20 (12.9)	126 (81.3)	9 (5.8)
	2	152	148	18 (11.8)	116 (76.3)	14 (9.2)
	3	140	138	8 (5.7)	126 (90.0)	4 (2.9)
Pain/discomfort	1	155	155	40 (25.8)	105 (67.7)	10 (6.5)
	2	152	149	32 (21.1)	105 (69.1)	12 (7.9)
	3	140	138	29 (20.7)	102 (72.9)	7 (5.0)
Anxiety/depression	1	155	155	27 (17.4)	119 (76.8)	9 (5.8)
	2	152	149	18 (11.8)	121 (79.6)	10 (6.6)
	3	140	138	18 (12.9)	111 (79.3)	9 (6.4)

*EQ-5D* EuroQol 5-dimensions questionnaire, three-level, *FAS* full analysis set, *N* number of subjects treated, *Nobs* number of observed cases

The proportion of subjects with ‘improvement’ was ≥10% greater than that with ‘worsening’ of their condition for Mobility in injection cycle 1, Pain/discomfort for all injection cycles, and Anxiety/depression in injection cycle 1. At 4 weeks post-injection, the majority of subjects had ‘no change’ in the rating of their condition within each injection cycle for individual EQ-5D dimensions (Table 2).

**Fig. 2:**
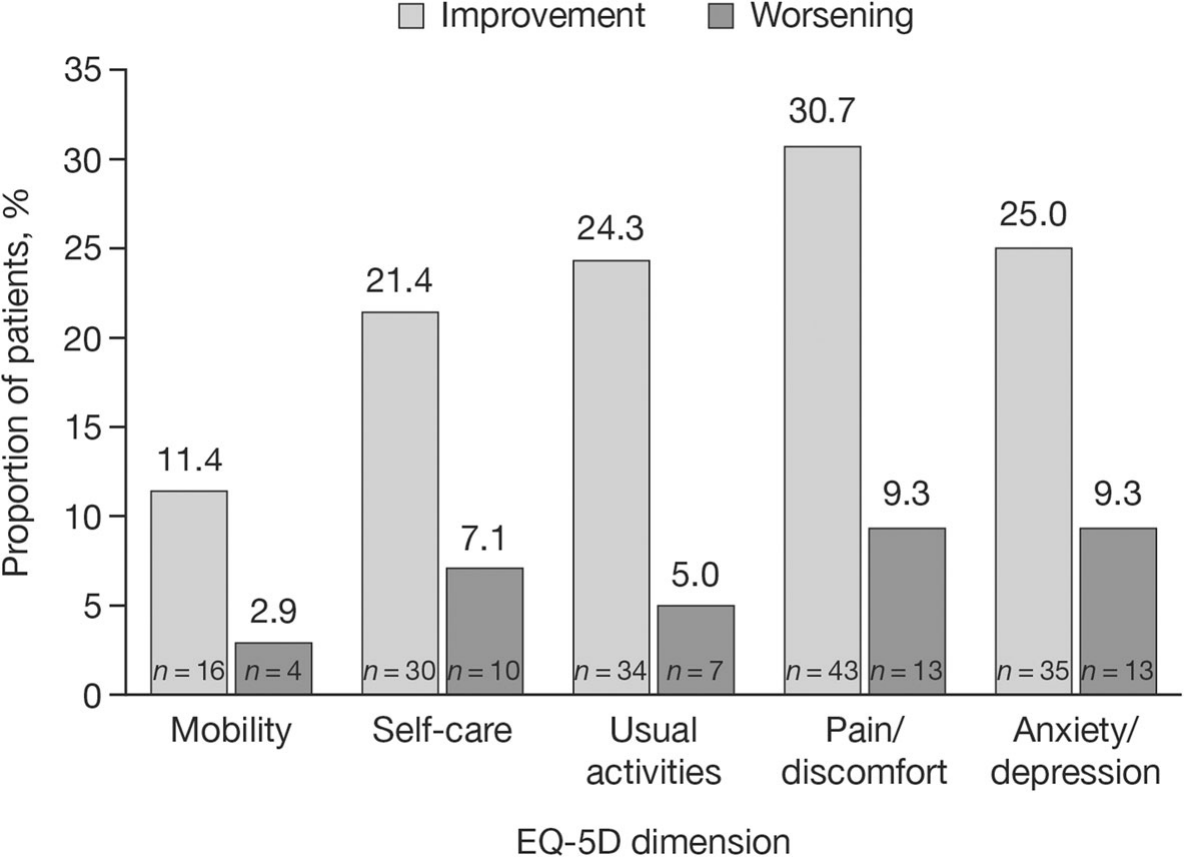
Subjects with improvement or worsening of EQ-5D ratings in all dimensions (FAS). Time points: from study baseline to the end of injection cycle 3 (*N* = 138). *EQ-5D*, EuroQol 5-dimensions questionnaire, three-level; *FAS*, full analysis set; *n*, number of observed cases

From the study baseline to the end of injection cycle 3, the proportion of subjects with improvements in EQ-5D ratings was greater than the proportion of subjects with worsening of ratings for all dimensions: Mobility, 11.4% versus 2.9%; Self-care, 21.4% versus 7.1%; Usual activities, 24.3% versus 5.0%; Pain/discomfort, 30.7% versus 9.3%; Anxiety/depression, 25.0% versus 9.3% (Fig. 2). The proportions of subjects rating their condition as ‘normal’, ‘moderate impairment’, or ‘severe impairment’ for each dimension at study baseline and the end of injection cycle 3 are shown in Table 3.

**Table 3:** EQ-5D dimension score frequency at study baseline and end of cycle 3 (FAS)

Dimension	Visit	*N*	Nobs	Frequency, *n* (%) subjects		
				1 Normal condition	2 Moderate impairment	3 Severe impairment
Mobility	Baseline	155	155	12 (7.7)	139 (89.7)	4 (2.6)
	End of injection cycle 3	140	137	23 (16.4)	112 (80.0)	2 (1.4)
Self-care	Baseline	155	155	30 (19.4)	96 (61.9)	29 (18.7)
	End of injection cycle 3	140	137	33 (23.6)	93 (66.4)	11 (7.9)
Usual activities	Baseline	155	155	11 (7.1)	113 (72.9)	31 (20.0)
	End of injection cycle 3	140	137	22 (15.7)	103 (73.6)	12 (8.6)
Pain/discomfort	Baseline	155	155	53 (34.2)	85 (54.8)	17 (11.0)
	End of injection cycle 3	140	137	72 (51.4)	60 (42.9)	5 (3.6)
Anxiety/depression	Baseline	155	155	72 (46.5)	69 (44.5)	14 (9.0)
	End of injection cycle 3	140	137	82 (58.6)	53 (37.9)	2 (1.4)

*EQ-5D* EuroQol 5-dimensions questionnaire, three-level, *FAS* full analysis set, *N* number of subjects treated, *Nobs* number of observed cases

**Fig. 3:**
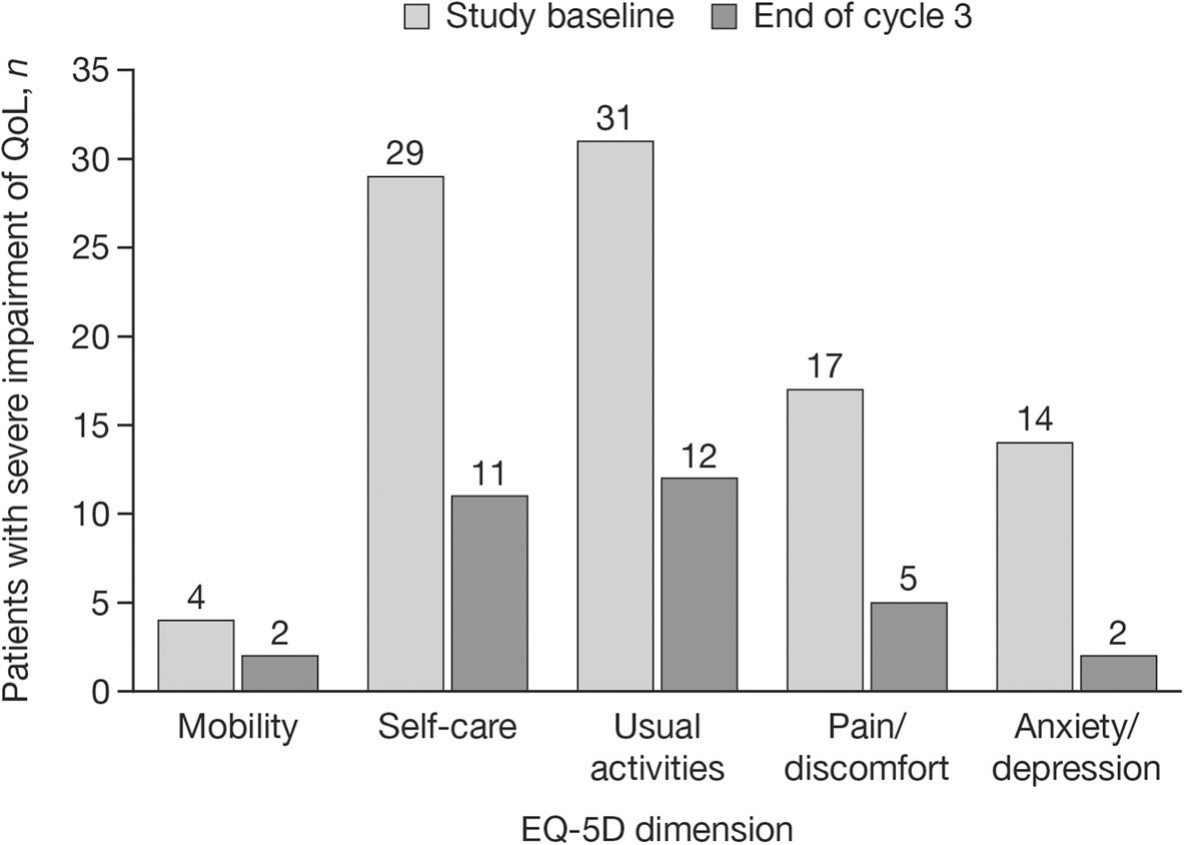
‘Severe impairment’ of QoL at study baseline and end of cycle 3 (FAS). EQ-5D, EuroQol 5-dimensions questionnaire, three-level; FAS, full analysis set; *n*, number of observed cases; QoL, quality of life

Across all dimensions, compared with study baseline, there was an increase in the proportion of subjects rating their condition as ‘normal’ at the end of injection cycle 3. This improvement was > 10% for Pain/discomfort (34.2% at baseline to 51.4% at end of injection cycle 3) and Anxiety/depression (46.5% at baseline to 58.6% at end of injection cycle 3). The proportion of subjects with ‘severe impairment’ was reduced by ≥46% for all EQ-5D dimensions at the end of injection cycle 3 compared with study baseline; however, this condition rating applied to a small proportion of subjects overall (≤ 20% at baseline for all dimensions; Table 3 and Fig. 3).

## Discussion

TOWER was the first prospective clinical study to demonstrate that dose escalation with total body doses of incobotulinumtoxinA up to 800 U enables treatment in a greater number of muscles/spasticity patterns, without compromising safety or tolerability [20]. Consistent with the observed improvements in muscle tone and better goal attainment associated with dose escalation from 400 to 800 U of incobotulinumtoxinA presented previously [20], we demonstrated the beneficial effects of incobotulinumtoxinA on QoL among this study population. There were sustained improvements in QoL across all three injection cycles, as well as improvements in self-assessed health status (EQ-5D VAS) across injection cycles. Among those subjects who had a change in the rating of their condition, at the end of injection cycle 3 a greater proportion of subjects had improvement of ratings from injection cycle baseline than worsening of ratings for all dimensions of the EQ-5D. Similarly, the proportion of subjects with a rating of ‘normal condition’ in each of the five EQ-5D dimensions was greater and the proportion with ‘severe impairment’ to QoL was lower at the end of injection cycle 3, compared with ratings at study baseline. Of note, there was a ≥ 46% decrease in the proportion of subjects with ‘severe impairment’ for all five EQ-5D dimensions by the end of injection cycle 3. However, the numbers of subjects with ratings of ‘severe impairment’ were low, particularly for the Mobility dimension. The factors that may identify which subjects will experience QoL improvements that can be measured using EQ-5D are currently unknown. Furthermore, as this was an exploratory trial without a parallel fixed-dose comparator arm or placebo control, these QoL results are descriptive; randomized controlled studies are required to confirm the findings.

Few studies have investigated the effect of BoNT-A treatment on subjects’ QoL [1, 3]. In a non-interventional study, incobotulinumtoxinA in combination with conventional therapy (oral antispasticity medication, physiotherapy, and occupational therapy) significantly improved QoL (assessed by 12-Item Short Form Health Survey score) compared with baseline during 1-year treatment of post-stroke upper-limb spasticity [12]. The TOWER study is, to our knowledge, the first to provide some evidence of an improvement in QoL using escalating doses of BoNT-A for the simultaneous treatment of upper- and lower-limb spasticity. Dose escalation allowed the physician the flexibility to increase the dose per muscle within the defined dose range, and enabled the treatment of more muscles and clinical patterns according to the needs and treatment goals of the individual subject [20], and this may have contributed to improvement in QoL.

The EQ-5D has previously been validated in subjects following stroke [28], who form the largest proportion of the TOWER study population. Differences in EQ-5D scores between stroke survivors with and without spasticity met the minimal clinically important differences previously established for other validated diseases, potentially supporting the use of EQ-5D as a measure of QoL in this population [4]. However, the use of EQ-5D may be considered a limitation of this study, since it is not validated for use in spasticity.

The three-level EQ-5D, which was the only version available at the outset of this study, may lack the sensitivity required to capture incremental but meaningful changes in QoL in these subjects. For example, a previous randomized clinical trial of BoNT-A that used the EQ-5D questionnaire to measure QoL in subjects with upper-limb spasticity due to stroke did not demonstrate any significant improvement in the Mobility, Self-care, or Usual activities dimensions up to 12 months post-treatment [29]. Although significant improvements were observed at some time points for the Pain/discomfort and Anxiety/depression dimensions, the magnitude of these changes was negligible and the clinical significance was unclear [29]. Pain/discomfort may be most successfully alleviated with high-dose treatment. Therefore, it may be noteworthy that Pain/discomfort and Anxiety/depression were also the dimensions for which we observed the highest frequency of subjects with improvement and the greatest proportion of subjects rating their condition as ‘normal’ at the end of injection cycle 3. While these findings might suggest that treatment with BoNT-A has a positive impact on these aspects of QoL, it is also possible that these are the dimensions most likely to be influenced by the clinical trial environment where subjects are receiving highly structured spasticity management from a specialist team. Notably, the comparatively low proportion of subjects who experienced an improvement in Mobility, Self-care, and Usual activities is likely to reflect a high degree of underlying motor deficits in this chronic population, as demonstrated by the high REPAS and DAS scores at study baseline. This suggests that subjects experiencing painful spasticity, but with sufficient underlying motor function, may experience greater QoL improvements with high-dose incobotulinumtoxinA, compared with subjects with significant functional impairment.

Interestingly, the greatest proportion of subjects with improvements in all EQ-5D dimensions occurred following treatment with 400 U in the first injection cycle, with sustained improvement in subsequent injection cycles, suggesting a ceiling effect, which has also been observed in previous studies using the three-level EQ-5D [30]. Future studies of QoL in subjects with spasticity may benefit from use of the five-level EQ-5D [31], which incorporates additional terms to describe health status and is expected to offer better sensitivity and discriminatory capacity than the EQ-5D [31, 32]. Novel tools, such as the Spasticity-Related Quality-of-Life Tool (SQoL-6D), are in development specifically for spasticity and may also be of value for future research once validated [33]. Given the limited assessment tools available, it is unsurprising that there is a paucity of data on QoL in subjects with chronic spasticity. In particular, the lack of normative QoL data for subjects with upper- and lower-limb spasticity is a major limitation when interpreting changes in QoL as a result of BoNT-A intervention. It is intended that the results reported here will serve as a valuable starting point for further research in this field.

## Conclusions

The safety and efficacy of BoNT-A formulations for the treatment of upper- and lower-limb spasticity are well established, and these formulations form a key component of the multidisciplinary management of this condition [9–19, 34, 35]. A subject-centered approach to management of spasticity, as shown for incobotulinumtoxinA, is important to help subjects achieve their individual goals and also improve their QoL [6]. Consistent with previous reports [21, 36–38], results of the TOWER study showed that treatment with total body doses of incobotulinumtoxinA up to 800 U is well tolerated, and no new safety concerns were identified for higher cumulative doses [20]. Using higher doses of incobotulinumtoxinA enables simultaneous treatment of more clinical patterns of upper- and lower-limb spasticity that is very likely to also contribute to improvement in multiple aspects of subjects’ QoL, supporting its potential role in the rehabilitation of subjects with multifocal upper- and lower-limb spasticity.

## Supplementary information

**Additional file 1: Fig. S1.** Subject disposition.

## Data Availability

Additional data for the TOWER study are available to researchers, and can be found in the ClinicalTrials.gov record (NCT01603459).

## Abbreviations

BMI: Body mass index
BoNT-A: Botulinum neurotoxin type A
BoNT-B: Botulinum neurotoxin type B
CI: Confidence interval
DAS: Disability Assessment Scale
EPAS: Resistance to Passive Movement Scale
EQ-5D: EuroQol 5-dimensions questionnaire, three-level
FAS: Full analysis set
FEV_1_: Forced expiratory volume in 1 s
INR: International normalized ratio
QoL: Quality of life
SD: Standard deviation
SQoL-6D: Spasticity-Related Quality-of-Life Tool
TOWER: The Titration study in lOWer and uppER limb spasticity
VAS: Visual analog scale
