# Supplementary figures and images for: Quality of life in subjects with upper- and lower-limb spasticity treated with incobotulinumtoxinA
Source: Health Qual Life Outcomes. 2020 Mar 4;18:51. doi: 10.1186/s12955-020-01304-4 (PMC7055124; doi:10.1186/s12955-020-01304-4)

Additional file 1

Fig. S1 Subject disposition

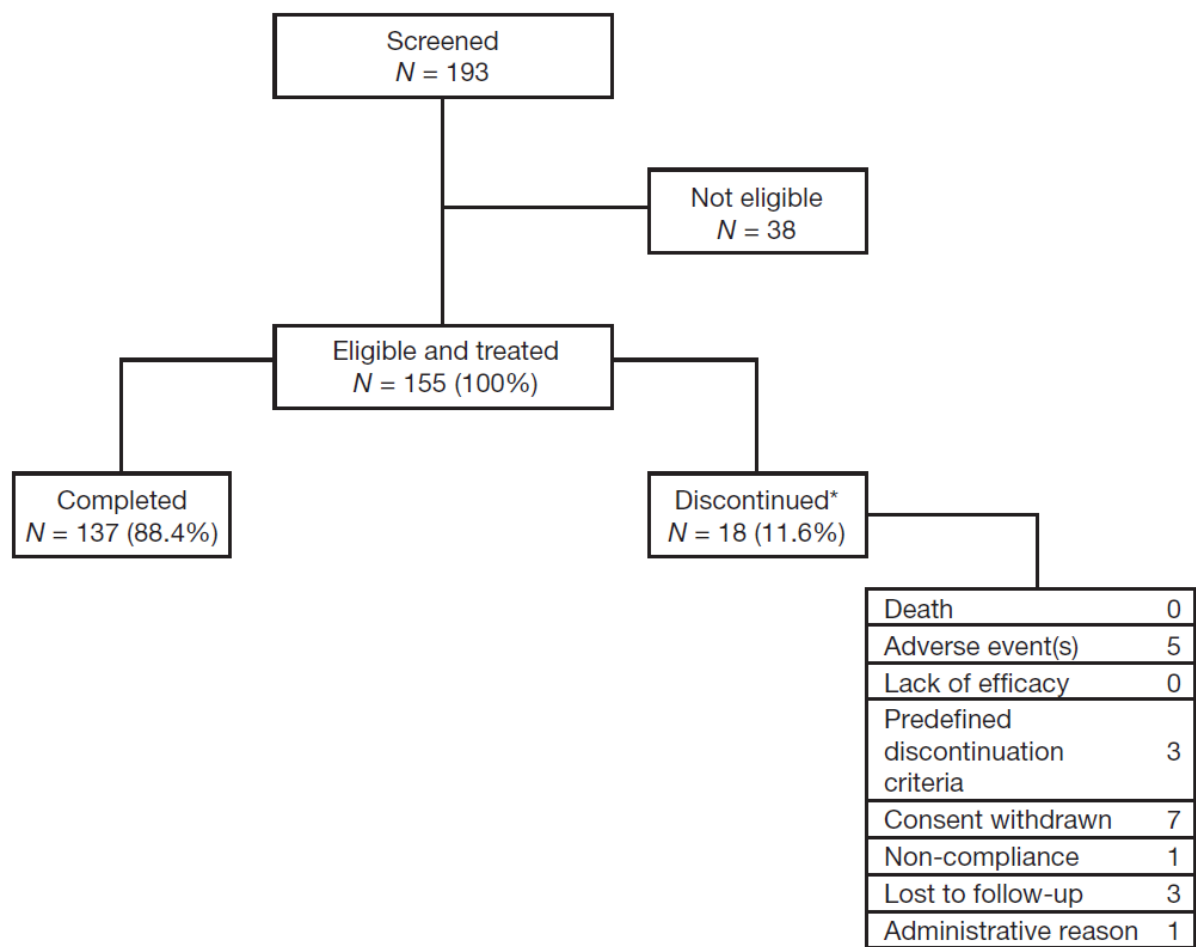

\*multiple entries possible

Supplement: Supplementary file 1 — Additional file 1: Fig. S1. Subject disposition. [file 12955_2020_1304_MOESM1_ESM.pdf]
